# Movement Matters! Understanding the Developmental Trajectory of Embodied Planning

**DOI:** 10.3389/fpsyg.2021.633100

**Published:** 2021-04-28

**Authors:** Lisa Musculus, Azzurra Ruggeri, Markus Raab

**Affiliations:** ^1^Department of Performance Psychology, Institute of Psychology, German Sport University Cologne, Cologne, Germany; ^2^iSearch, Max Planck Research Group, Max Planck Institute for Human Development, Berlin, Germany; ^3^TUM School of Education, Technical University Munich, Munich, Germany; ^4^School of Applied Sciences, London South Bank University, London, United Kingdom

**Keywords:** embodied cognition, planning, lifespan, development, children

## Abstract

Human motor skills are exceptional compared to other species, no less than their cognitive skills. In this perspective paper, we suggest that “movement matters!,” implying that motor development is a crucial driving force of cognitive development, much more impactful than previously acknowledged. Thus, we argue that to fully understand and explain developmental changes, it is necessary to consider the interaction of motor and cognitive skills. We exemplify this argument by introducing the concept of “embodied planning,” which takes an embodied cognition perspective on planning development throughout childhood. From this integrated, comprehensive framework, we present a novel climbing paradigm as the ideal testbed to explore the development of embodied planning in childhood and across the lifespan. Finally, we outline future research directions and discuss practical applications of the work on developmental embodied planning for robotics, sports, and education.

## 1. Introduction

Humans start moving already in the womb, when they are just a few weeks old (Rahilly and Gardner, 1975), and develop the most sophisticated motor skills throughout the first years of life. Indeed, very young children are already able to thread a needle, build complex LEGO spaceships, and eat with chopsticks. Eventually, some humans reach a motor mastery that enables them to perform complicated heart surgeries or execute a triple twisting-double in gymnastics (the *Biles II*). Over the first years of childhood, humans' cognitive skills reach similar levels of extreme sophistication. Children can memorize entire poems, learn complex game rules, and manage to perform several tasks at the same time—eating, playing with a doll, binge-watching TV, and following a conversation simultaneously. Most adults can stay focused on the street and ignore irrelevant information while driving, and some are eventually able to control air traffic, play chess, and solve a Rubik's cube blindfolded. While newborns are far away from mastering any of these sophisticated motor or cognitive tasks, they will eventually acquire these or comparably complex skills throughout development. How do motor and cognitive development interact and impact each other? In this perspective paper, we argue that “movement matters!,” implying that motor development is a crucial driving force of cognitive development, much more impactful than previously acknowledged. In this regard, we argue that to fully understand and explain developmental changes, it is necessary to consider the interaction of motor and cognitive skills from a developmental embodied cognition perspective.

In what follows, we first introduce developmental embodied cognition. Second, we exemplify our argument by introducing the concept of “embodied planning” integrating the motor and cognitive perspectives on planning and derive developmental predictions. Third, we present a novel climbing paradigm as the ideal testbed to capture and explore the development of embodied planning during childhood and across the lifespan. Finally, we outline future research directions and discuss practical applications of the work on developmental embodied cognition, and in particular of embodied planning, for robotics, sports, and education.

## 2. A Developmental Embodied Cognition Perspective: Why Movement Matters!

Hundreds of studies have documented the influence of sensorimotor manipulations on cognition, such as abstract spatial and temporal presentation (Loeffler et al., 2016), memory retrieval (Dijkstra et al., 2007), number processing (Fischer et al., 2004), or decision making (Lepora and Pezzulo, 2015). A central tenet of embodied cognition is that cognitive skills are “deeply routed” (Wilson, 2002) in the body, sensorimotor experiences, and the environment (Fischer and Coello, 2016). In this regard, it is already clear how crucial it is to consider the body as well as sensorimotor experiences and motor skills when trying to understand cognitive skills (Glenberg et al., 2013). From an embodied point of view, the interaction between sensorimotor and cognitive skills is theoretically predicted and has empirically been shown to be bidirectional and dynamic, although only a few studies have addressed the influence of cognition on sensorimotor processes (Engel et al., 2013).

Most sophisticated motor and cognitive skills are learned throughout development. Previous work already proposed that motor skills are the foundation of cognitive development (Ridler et al., 2006; Koziol et al., 2012; Gottwald et al., 2016) and provide the basis for learning (Adolph and Hoch, 2019). Different lines of research support this claim by showing that cognitive changes are associated with bodily changes (Hommel and Kibele, 2016), and that cognitive performance benefits from instructions activating bodily experience through body analogies (Pouw et al., 2016) or from acting (Lozada and Carro, 2016). Gottwald et al. (2016) recently demonstrated an association between motor control and executive functions in infants, finding that prospective motor control during reaching (i.e., peak velocity of the first movement) was correlated to inhibition and working memory. The potential magnitude of the impact of early motor skills on cognition is further demonstrated by a study from Ridler et al. (2006) showing that infants' gross motor skills predicted executive functions in adulthood. In particular, those infants who managed to stand and walk earlier in their life had superior cognitive skills in their thirties and showed higher gray-matter density in motor areas (Ridler et al., 2006).

There are several reasons why we argue that an *embodied cognition perspective* is extremely fruitful, if not necessary, to understand the developmental trajectory of motor–cognitive interactions. Together, bodily growth and the acquisition of new motor skills enable and support children's learning, acting as a driving force of cognitive development (Adolph and Hoch, 2019). Across the lifespan, human experience consists of an alternation of phases characterized by rapid change and phases of greater stability. In phases of change, embodiment effects can be captured particularly well: When our bodies change more dramatically and motor skills improve notably, as during infancy, childhood, and adolescence (Portella et al., 2017; Adolph and Hoch, 2019), or in older age (Cole et al., 2019), the impact of these changes on cognitive processes can be more easily scrutinized, and a time-ordered, causal direction can be tested.

In this perspective paper, we support this claim by focusing on the development of planning throughout childhood. Developmental research on motor and cognitive planning exists unnoticed from each other. However, we argue that both motor and cognitive components need to be considered jointly to understand the developmental trajectory of planning and its relevance for actions in the real world, beyond controlled lab environments.

## 3. Embodied Planning: Integrating Theoretical and Methodological Approaches

### 3.1. Motor Planning

Motor planning is defined as the ability to organize motor behavior to accomplish an anticipated goal-directed action. By definition motor planning processes depend on goal proximity: To adjust motor behavior to an imminent goal is referred to as first-order motor planning, whereas adjusting to subsequent goals is referred to as second-order motor planning (Rosenbaum et al., 2012; Domellöf et al., 2020). In tasks assessing motor planning, participants are required to first plan and then execute a motor sequence, during which the motor system needs to be controlled and can be adjusted. Classic motor planning tasks, used with children as well as with adults, are the bar-transport task (Knudsen et al., 2012), the overturned-glass task (Adalbjornsson et al., 2008; Knudsen et al., 2012), and the handle rotation task (Craje et al., 2010). In the bar-transportation task, for example, children are asked to insert a bar into a small opening of a box. In the trials requiring two-steps planning, children need to grasp the bar with a (rather uncomfortable) thumb-down grip, then rotate the bar by 180 and conclude the insertion in the much more comfortable thumb-up position. Second-order motor planning is somewhat limited until the age of 6 years (Benson et al., 2018), although improvements between the age of 3–6 years have been reported (Knudsen et al., 2012). At the age of 10, children usually reach adult-like motor planning skills (Benson et al., 2018).

### 3.2. Cognitive Planning

Cognitive planning is defined as the ability to think about action sequences in advance, thus approaching a task in an organized, strategic, and efficient manner (Anderson, 2002; Best et al., 2009), and is considered an essential requirement of goal-directed behavior. In tasks assessing cognitive planning, participants are required to plan ahead, evaluate, implement, and then potentially modify a sequence of actions (Best et al., 2009). Classic cognitive planning tasks used across the lifespan are the Tower of London (Bull et al., 2004) or maze navigation tasks (Völter and Call, 2014), where children are asked to move a reward through multiple levels of a maze. For planning which route to take, children have to consider whether the passages on the next levels are open or closed. Being a higher-order cognitive function that relies on working memory and inhibition (Best and Miller, 2010; McCormack and Atance, 2011), cognitive planning skills emerge rather late. For instance, 4-year-olds can plan only one step ahead, that is, considering only the configuration of passages onto the immediate next level of the maze, whereas 5-year-olds can plan two steps ahead (Völter and Call, 2014). Planning complex action sequences develops in late childhood or adolescence (Best et al., 2009), and only by the age of 15 children reach adult-like cognitive planning skills (Huizinga et al., 2006). Besides, even adults do not always plan as efficiently as possible (Meder et al., 2019).

### 3.3. Theoretical Integration of Motor and Cognitive Planning

The developmental trajectories of motor and cognitive planning have been studied separately. However, the interaction of motor and cognitive planning in general, and in particular during development, is to date not well understood. Specifically, cognitive planning has largely been investigated with tasks entailing no (or to a very low degree) motor planning or ignoring the motor component altogether. In this paper, we introduce the concept of “embodied planning,” which integrates perspectives and methods from cognitive and movement sciences.

**Figure 1:**
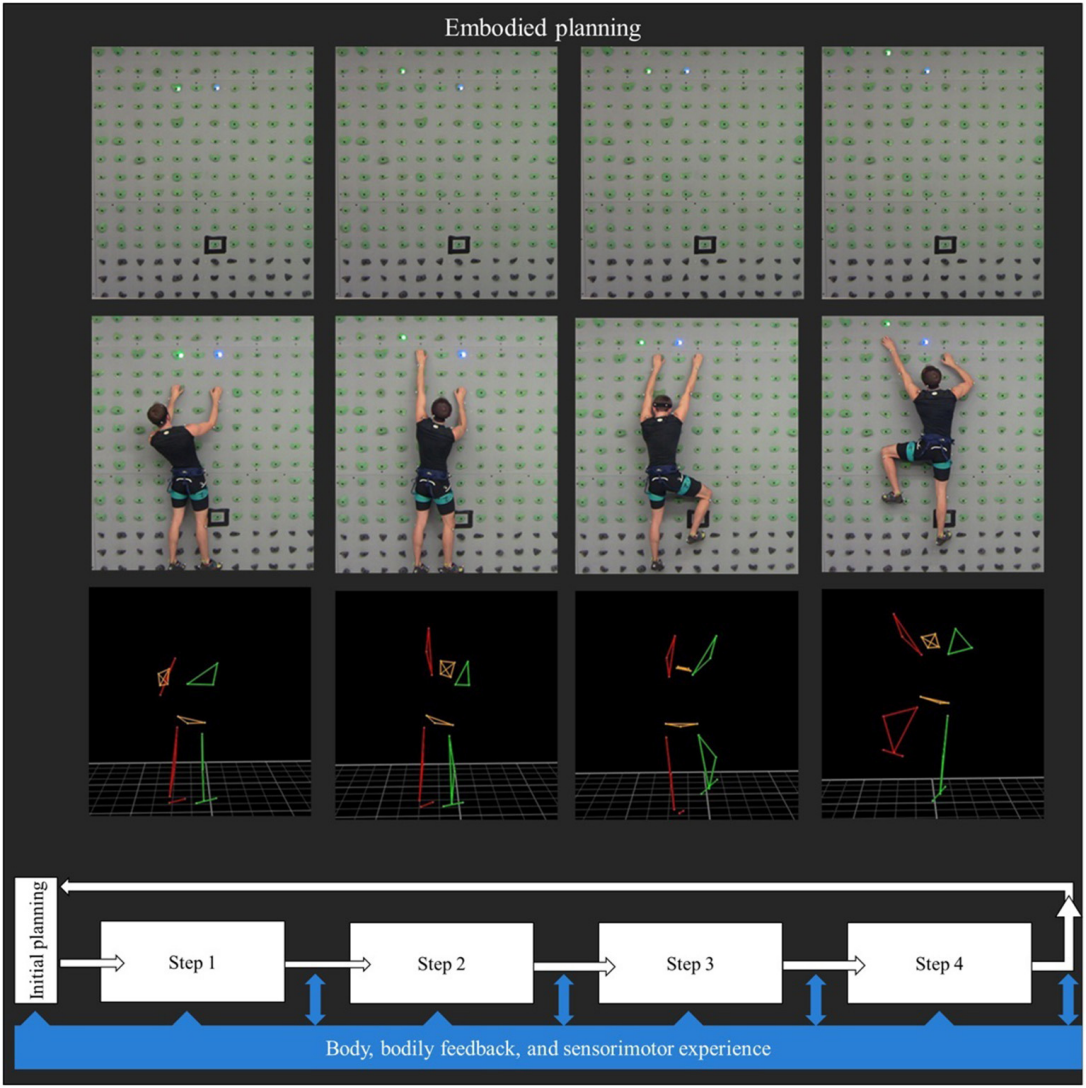
Embodied planning in a climbing paradigm. The figure depicts four steps of embodied planning during a goal-directed climbing task. At the bottom, the embodied-planning concept is modeled as a dynamic, continuous feedback-loop between motor and cognitive planning. The two upper panels show an interactive climbing wall (2.40–3.60 m) with touch-sensitive climbing holds, which can light up in different colors and capture reaction times (i.e., ClimbLing system). The climbing system is synchronized to a movement-analysis system (Vicon; 10 infrared cameras at 119.88 Hz, VICONTM, Oxford, UK), which captures full-body movement kinematics as indicated by the stick figure of the body.

Embodied planning involves cognitive planning, which takes place before starting the execution of a motor-cognitive task, but assumes that one's bodily state, physical constraints, and (previous) motor experience provide cues for the planning process (cf. similar models in choice: embodied choices; Cisek and Pastor-Bernier, 2014; Wyer, 2016; Raab, 2017). Therefore, cognitive planning is *guided* by the awareness of how exactly a step can be executed through coordination of the motor systems (Raab et al., 2005), and by the feedback from the motor implementation at any previous step. Accordingly, embodied planning can be conceptualized as a dynamic, continuous feedback-loop between motor and cognitive planning in a goal-directed planning task, as illustrated in Figure 1.

### 3.4. Developmental Predictions

Based on the theoretical notion of embodied planning and the existing developmental evidence reviewed above, we can derive predictions at different levels of specificity. In general, we expect that the development of embodied planning across childhood will follow a nonlinear trajectory (Best et al., 2009), depicting stronger changes during infancy, early childhood, and adolescence—phases of more pronounced bodily change. More specifically, we predict that bodily changes will affect the motor aspects of planning first, the improvement of which will promote cognitive planning. Previous developmental findings support this claimed chronology, showing that although the motor planning skills reach maturity already around 10 years of age, cognitive planning develops way beyond age 10, reaching adult-like sophistication only in late adolescence. This developmental chronology might suggest that, indeed, cognitive planning skills are preceded by, fostered by, and build on improved motor planning skills.

Zooming in on this proposed developmental trajectory of embodied planning right on the onset of bodily change, we would predict that bodily changes first entail learning new motor skills and adapting already acquired ones, which in turn would trigger changes in motor planning. Changes in motor planning might then impact cognitive planning performance negatively, taking away additional resources required to fulfill the new motor planning demands (cf., embodied-cognitive-load hypothesis Warburton et al., 2013; for a summary of developmental work, see Berger, 2010; Berger et al., 2018). However, once the new motor skills are mastered and the corresponding improvements in motor planning are assimilated, cognitive planning might also improve, benefiting from motor planning efficiency.

### 3.5. Methodological and Technological Advances

The integrative theoretical approach and developmental predictions we propose have important methodological consequences. To be able to empirically capture and study the developmental trajectory of embodied planning, new designs, tasks, and measures have to be developed and implemented.

First, experimental *designs* should allow monitoring intra-individual changes and inter-individual differences throughout development. Intra-individual changes can be tested in longitudinal designs (Musculus et al., 2019), as well as in intervention and training studies (Harbourne and Berger, 2019). Inter-individual differences can be analyzed with cross-sectional comparisons of different age groups (Berger et al., 2015; Benson et al., 2018). Ideally, longitudinal, intervention, or training designs should be combined with cross-sectional age-group comparisons to best disentangle the developmental dynamics from individual differences and control for potentially confounding variables.

Second, the planning *tasks* should exert similar demands on both the motor and the cognitive systems, that is, both motor and cognitive planning skills should be required to solve the task, and to a similar extent. Additionally, the movements executed for and/or during the task need to be *task relevant* (Wilson and Golonka, 2013), not simply constituting a random motor response (pressing a button) that could be potentially interchanged with any other simple motor reaction (pulling a lever). Third, the *measures* implemented should be able to capture motor and cognitive interactions in embodied planning, ideally online. This is why we propose to combine movement analysis with reaction times. In developmental research, movement analysis has been proven an objective, fine-grained method to assess motor development (van Schaik and Dominici, 2020). In particular, marker-based motion tracking systems can provide accurate measures of motor processes (van Schaik and Dominici, 2020). With marker-based systems, the position of children's joints can be tracked with specific camera systems while they move. During task execution, movement trajectories in 3D space can be captured (i.e., kinematics, see Figure 1). Although developmental studies exist that analyzed children's kinematics and response times (Domellöf et al., 2020), only a few combined the measures to explore the interaction between motor and cognitive skills (for an exception, see Gottwald et al., 2016). Domellöf et al. (2020) analyzed age-related differences in the spatiotemporal segmentation of the movement path for the wrist, index finger, and object during a peg fitting task. Their kinematic analyses provided a more detailed picture of the time course of motor planning and revealed developmental differences: While adults rotated the peg during transport, 6–10 year-old children most often did so only after reaching the goal. Integrating kinematics to the previously used cognitive measures allowed to capture that children did not engage in planning ahead as much as adults did, thus demonstrating less efficient motor planning. Along the same lines, the work of Gottwald et al. (2016) revealed that the peak velocity of infants' first movement in a prospective planning task captured the extent of their motor planning, which was related to their higher-order cognitive control. These studies highlight how the combined analysis of motor and cognitive processes is necessary to capture embodied-planning development in childhood.

To exemplify the design, task, and measurement requirements presented, we introduce a novel climbing paradigm to capture the developmental dynamics of embodied planning accordingly.

## 4. Climbing as a Testbed for Embodied Planning

Climbing to a predefined goal naturally involves embodied planning, requiring both complex cognitive (Cascone et al., 2013) and motor planning (Testa et al., 2003) skills. To successfully climb, one needs to plan *which* route to climb—which holds to use, and in what order—as well as *how* to execute the route (Raab, 2017). In particular, climbers need to consider their body constraints and the motor skills required to execute every single move. Then, during climbing, continuous sensorimotor (e.g., of muscles, hands, feet) and cognitive (e.g., which hold should I use next?) feedback fuels back, dynamically, into the ongoing planning process.

Crucially, climbing tasks are perfectly suitable to be used with a very wide age range, as they can be performed (and with great fun) by young children and adults (Croft et al., 2018). Indeed, children have a natural tendency to climb all sorts of things, from home furniture to playground constructions, to trees. Further, experimental climbing tasks can be used to explore body and action boundaries (van Knobelsdorff et al., 2020; Seifert et al., 2021), also in children (Croft et al., 2018): A recent study showed that 6- to 11-year-old children who were more accurate in judging their reaching capability (i.e., whether they were able to reach and grasp holds that are near or far away) completed more climbing routes successfully and did so faster (Croft et al., 2018).

Methodologically speaking, goal-directed climbing comprises motor and cognitive planning before the task and during execution. In experimental settings, climbing tasks can be easily adjusted and modified to manipulate cognitive and motor demands, such that motor planning but only little cognitive planning is required, or the other way around. In particular, cognitive planning could be minimized by guiding children through all steps (i.e., hold) along a predefined route. In particular, by using an interactive climbing wall (e.g., ClimbLing), one could indicate the next hold that children should use by lighting it up. To reduce cognitive planning to a minimum, the next hold would light up only after the previous hold has been touched (please refer to Figure 1). At the same time, motor planning could be reduced to a minimum by asking children to plan a route without executing it. In particular, children could be asked to plan a climbing route by just indicating to the experimenter which holds they would use using a laser pointer or on an app that displays the climbing wall. Thus, by carefully designing novel climbing tasks, motor and cognitive planning processes could be disentangled experimentally.

Given in regular climbing motor and cognitive planning constantly interact, the joint consideration of motor and cognitive measures in climbing experiments is warranted. From the motor side, anthropometric measures and movement analyses associated with climbing performance should be captured, such as weight (Mermier et al., 2000; Watts et al., 2003), height (Watts et al., 2003; Laffaye et al., 2016), body mass index (Laffaye et al., 2016), grip strength (Mermier et al., 2000), finger-tip strength (van Knobelsdorff et al., 2020), and Ape-index (Mitchell et al., 2011). For movement kinematics, spatial and temporal movement dimensions should be considered (Orth et al., 2016, 2017). In particular, the immobility–mobility ratio (IMR) and the geometric index of entropy (GIE) represent temporal-spatial indices capturing motor planning in climbing (Orth et al., 2017). Importantly, the movement data should be time-matched and related to cognitive measures during the task, such as the number of holds to indicate planning steps (Huizinga et al., 2006), the time used to complete the route, and the initial planning time used before starting task execution (Huizinga et al., 2006). Complementing these “classical” cognitive planning variables by capturing gaze patterns during initial route preparation (Seifert et al., 2017; van Knobelsdorff et al., 2020) and the time course of movement variability during route execution can provide a better picture of the planning strategies.

Together, due to the close connection of motor and cognitive planning during goal-directed climbing, the ongoing embodied-planning dynamics can be captured and the performance in previous planning steps can be related to the next and so forth. Climbing as a task is useful because completing a route is only possible by a sequence of embodied-planning steps. Therefore, the unfolding of motor and cognitive processes from initially planning a route through route execution can shed light on the interaction—embodied planning.

## 5. Future Research Directions and Potential Applications of Embodied Planning

Improvements in embodied planning across the lifespan can be seen as a goal on its own, or as a means to an end. On the one hand, research on embodied planning can support coaches and teachers in developing interventions that target and boost motor planning skills during the school-age years (Croft et al., 2018), e.g., by introducing climbing exercises as an integral part of PE curricula. On the other hand, embodied planning could also serve as a means to improve sports and academic performance. Recent results indicate that executive-function training through sports in school has positive effects with near transfer (Vazou et al., 2016). Embodied-planning training might be especially beneficial for students with lower academic skills (Pouw et al., 2016) or children with developmental delay (Harbourne and Berger, 2019). Indeed, children with a lower level of general mathematical abilities performed better in a physical problem-solving task when the instructions provided references to their bodies, for example when children had to mimic a lever with their arms (Pouw et al., 2016). Also children with developmental delays profited from a motor-based problem-solving intervention in terms of motor and cognitive gains (Harbourne and Berger, 2019). Thus, making use of the body, activating bodily representation, and providing (active) sensorimotor experience during embodied-planning exercises might constitute a promising intervention in sports and education.

Broadening the scope, we believe that experimental research on embodied planning could inform the development of more accurate and sophisticated models of human movement to be implemented in developmental robotics. First, fueling children's kinematics into a robotic system would support the development of robots able to move and plan *adaptively*. Second, developmental, cognitive, and movement scientists can profit from the robotic implementation of embodied-planning research to better understand developmental trajectories and individual differences in motor and cognitive development, and their interaction. Researchers could use simulations to test whether the way a person planned and executed their movements was functional or not. Along these lines, Ossmy et al. (2018) trained soccer-playing robots with kinematic walking data generated by infants during free play. The robots trained with a high variance of kinematic patterns won the simulated season of “RoboCup” (Ossmy et al., 2018) against robots trained with a low variance of kinematics. Similarly, aspects of motor planning in climbing such as the IMR or GIE could be evaluated. In detail, the kinematics of children climbing and of adults climbing can be fed into a robotics simulation. In the next step, the respective efficiency can be modeled, and, based on this, climbing training could be designed aiming at specific, efficient movement patterns.

## 6. Conclusion

In this perspective paper, we adopted a developmental embodied cognition perspective to argue that “movement matters!” for understanding the emergence and developmental trajectory of motor and cognitive skills, as cognitive development is fundamentally driven and constrained by motor development. We supported this claim by reviewing recent literature on motor and cognitive planning, so far researched in isolation. We then introduced the integrative theoretical concept of “embodied planning,” together with a novel climbing paradigm and related measures allowing to test it. Thereby, we intend to bridge the gap between the motor and cognitive disciplines. Overall, we highlighted the added value of embodied-planning research: Playful embodied-planning interventions could promote children's learning in the school setting and beyond. In the long run, embodied-planning research could contribute to the development of a new generation of adaptive robots.

## Data Availability Statement

The original contributions presented in the study are included in the article/supplementary material, further inquiries can be directed to the corresponding author/s.

## Author Contributions

LM drafted the article, which was edited by and discussed with AR and MR. All authors contributed to the development of this perspective and agree to be accountable for the content of the work.

## Conflict of Interest

The authors declare that the research was conducted in the absence of any commercial or financial relationships that could be construed as a potential conflict of interest.
